# Viral load and antibody boosting following herpes zoster diagnosis

**DOI:** 10.1016/j.jcv.2018.03.010

**Published:** 2018-06

**Authors:** Charlotte Warren-Gash, Harriet Forbes, Peter Maple, Mark Quinlivan, Judith Breuer

**Affiliations:** a Faculty of Epidemiology & Population Health, London School of Hygiene and Tropical Medicine, London, UK; b Virus Reference Department, Health Protection Agency (now Public Health England) Centre for Infections, Colindale, London, UK; c Division of Clinical Neuroscience, Faculty of Medicine and Health Sciences, Queen’s Medical Centre, University of Nottingham, Nottingham NG7 2UH, UK; d Division of Infection & Immunity, University College London, London, UK

**Keywords:** Antibody, Varicella zoster virus, Herpes zoster, Viral load

## Abstract

**Background:**

Acute varicella zoster virus (VZV) replication in shingles is accompanied by VZV antibody boosting. It is unclear whether persisting virus shedding affects antibody levels.

**Objectives:**

To investigate the relationship between VZV viral load and antibody titres in shingles patients during six months following diagnosis and assess whether VZV antibody titre could discriminate patients with recent shingles from healthy population controls.

**Study design:**

A prospective study of 63 patients with active zoster. Blood samples were collected at baseline, one, three and six months to measure VZV DNA and IgG antibody titre. We compared VZV antibody titres of zoster patients and 441 controls.

**Results:**

In acute zoster, viral load was highest at baseline and declined gradually over the following six months. Mean antibody titres rose fourfold, peaking at one month and remaining above baseline levels throughout the study. Antibody levels at one, three and six months after zoster were moderately correlated with baseline but not subsequent viral load. Regarding use of antibody titres to identify recent shingles, to achieve 80% sensitivity, specificity would be 23.4%, 67.7%, 64.8% and 52.6%, at baseline, visit 2, 3 and 4 respectively, whilst to achieve 80% specificity, sensitivity would be 28.3%, 66.1%, 52.6%, 38.6%, at baseline, visit 2, 3 and 4 respectively.

**Conclusions:**

Clinical VZV reactivation boosted VZV antibody levels and the level of boosting was dependent upon baseline viral replication. While antibody titres could discriminate patients with shingles 1–6 months earlier from blood donor controls, there was a large trade-off between sensitivity and specificity.

## Highlights

• Varicella zoster virus reactivation causes serum antibody boosting.
• Antibody titres at 1, 3 and 6 months following zoster diagnosis reflect baseline varicella zoster virus load.
• Antibody titres could discriminate patients post shingles from healthy blood donors, 1–6 months after shingles. However, to achieve a sensitivity of 80%, the specificity is between 55 and 70%, whilst to achieve 80% specificity, the sensitivity is between 35 and 70%.

## 1 Background

Primary infection with varicella zoster virus (VZV) causes chickenpox, following which the virus establishes latency. It reactivates in up to 25% of individuals to cause the painful dermatomal rash known as shingles (herpes zoster). During chickenpox or shingles, viral DNA is detectable in skin lesions, blood and saliva [[1], [2]]. Viral replication is accompanied by boosting of VZV antibodies consistent with antigenic, or endogenous, boosting. Few data exist, however, confirming the relationship between viral load and antibody titres during, and following, acute clinical VZV disease.

The extent to which the presence of persisting viral DNA in blood or saliva indicates active viral replication likely to induce an immune response is also unclear. Immunocompetent children with chickenpox clear viral DNA rapidly so that it is no longer detectable two weeks after the rash has healed [3]. In contrast, VZV DNA has been detected in blood for up to 6 months following shingles, albeit with falling loads [4]. Asymptomatic shedding of VZV in saliva occurs more frequently in individuals who are immune disadvantaged [[5], [6], [7]]. Better understanding of the spectrum of VZV reactivation is needed to inform use of biological markers of VZV reactivation in research.

## 2 Objectives

We aimed to investigate the relationship between VZV DNA levels and antibody titres by following acute shingles patients over 6 months, and to assess whether VZV antibody titre could discriminate patients with recent shingles from population controls for future research.

## 3 Study design

### 3.1 Study participants

Patients with shingles presenting to GPs in London between 2001 and 2003 were recruited consecutively for a prospective cohort study of disease burden, clinical and laboratory indices of zoster (described elsewhere) [4]. Diagnosis was confirmed through detection of VZV DNA from vesicle fluid by PCR in patients with clinically-suspected zoster. Patients completed a baseline survey that included demographic information, history of chickenpox and previous shingles episodes, immune status (including underlying illnesses and current treatment) and detailed information about the shingles episode (timing, symptoms, medications). Blood samples were taken at baseline, one, three and six months to measure IgG antibody titre and viral load. Blood samples from healthy blood donors from a single time-point were also collected.

### 3.2 Viral load and antibody measurements

Viral load was determined through detection and quantification of VZV DNA from whole blood. DNA extraction was performed using a QIAamp DNA blood mini kit (Qiagen Ltd, United Kingdom), with eluted DNA stored at −20 °C. VZV DNA was quantified using a real-time PCR assay, which had a sensitivity threshold of <10 VZV copies/μl(10). VZV IgG antibody titres were measured using a validated in-house time resolved fluorescence immunoassay [8]. Serum dilutions were tested in duplicate and the Europium counts obtained were interpolated against a standard curve of British Standard VZV antibody (NIBSC 90/690) covering the VZV IgG range 0.39–50 mIU/ml. Sera producing Europium counts outwith the curve were retested at appropriate dilutions. Duplicate results were averaged and multiplied by the dilution factor to obtain a final mean antibody level.

### 3.3 Statistical methods

We recoded implausible IgG values above the 95th blood donor percentile as missing (n = 23) and log transformed viral load and antibody titre to provide a normal distribution. We summarised the median, IQR and mean of the log transformed viral load and antibody titre at each time point. As there was no evidence of a non-linear association between logged mean viral DNA load and logged mean antibody titre we used Pearson’s correlation coefficients to investigate associations between these variables at the same and subsequent time points for shingles patients. These relationships were further explored using multivariable linear regression models. Potential confounding effects of age, sex, ethnicity, immunosuppression, days since rash onset, prodromal symptoms, disseminated rash and antiviral treatment were investigated using causal diagrams. Variables were retained if they were theoretically relevant confounders, and/or associated with both outcome and exposure at the 10% significance level using a forward selection approach.

To determine whether recent zoster could be identified from antibody levels, we undertook ROC analysis, comparing antibody levels in healthy controls with zoster patients. Antibody cut-off values (not on the log scale) to achieve 80% and 90% sensitivity or specificity, were calculated for each visit separately (along with the corresponding sensitivity or specificity), after adjusting for age and sex.

## 4 Results

The study comprised 63 patients with shingles, with a median age of 56 years (IQR 37–71 years) of whom 34 (54.0%) were male, and 441 blood donor controls (Table 1).

**Table 1:** Baseline characteristics of shingles patients and blood donors.

Variable	Group, frequency (%)	
	Shingles patients (N = 63)	Blood donors (N = 441)
Age, median (IQR), yrs	56 (37–71)	42 (29–51)
Sex		
Male	34 (54.0)	207 (46.9)
Female	29 (46.0)	234 (53.1)
Ethnicity^a^		
Afro-Caribbean	4 (6.3)	Data not available
Asian	3 (4.8)	
Caucasian	42 (66.7)	
Turkish-Caucasian	9 (14.3)	
Other	5 (7.9)	
Immunocompromised		
Yes	7 (11.1)	Data not available
No	56 (88.9)	
Rash age, days		
0–2	7 (11.1)	N/A
3–4	21 (33.3)	
5–6	22 (34.9)	
6+	13 (20.6)	
Prodromal symptoms		
Yes	46 (73.0)	N/A
No	17 (27.0)	
Disseminated rash		
Yes	7 (11.7)	N/A
No	53 (88.3)	
Missing	3 (–)	
Antiviral medication for shingles		
Yes	43 (68.3)	N/A
No	20 (31.7)	

a Modelled as ‘Caucasian’ versus ‘Non Caucasian’.

**Fig. 1:**
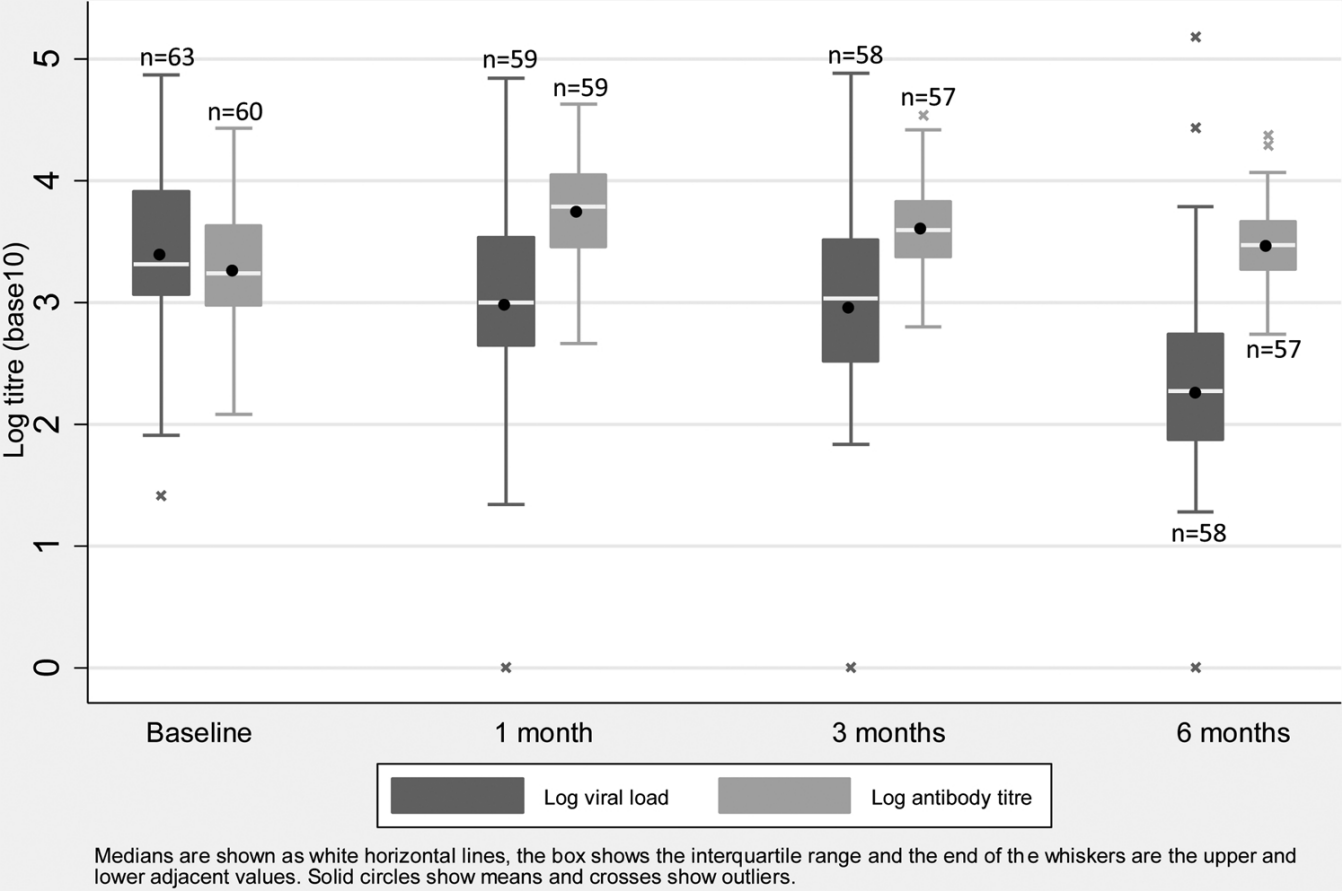
Log VZV viral load and antibody titres over time.

Viral load among shingles patients was highest at baseline and lowest at six months. Antibody titres rose from baseline to be maximal at one month then gradually declined, although titres remained elevated above baseline levels at six months (Fig. 1).

**Fig. 2:**
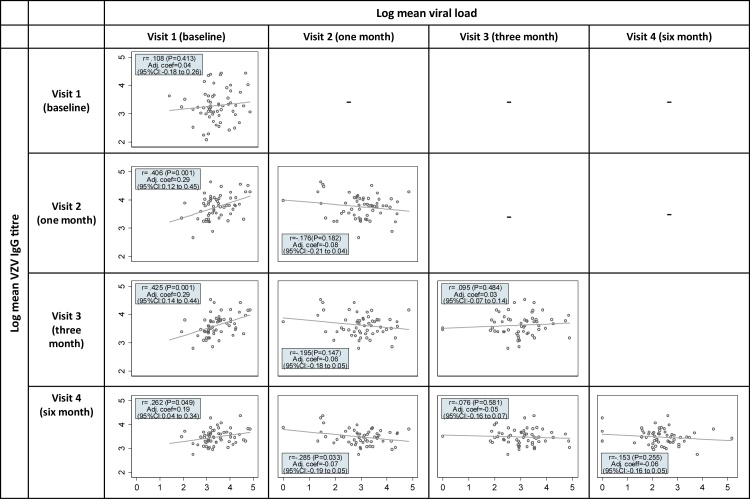
Association between log viral load and antibody titre, at the same and various time points: Pearson correlation coefficients and coefficients from multivariable linear regression models displayed. *Note*: Adjusted coefficients represent the effect of a one unit change in the variable value on the log mean antibody titre, adjusted for age, sex, ethnicity and immunosuppression status.

Viral load at baseline was positively associated with antibody titres at one, three and six months as shown in Fig. 2, although the strength of the associations were small to moderate. There was some evidence (P = .033) of a small negative correlation (r = −0.285) between viral load at one month and antibody titre at six months, but there were otherwise no significant associations between viral load measurements taken after baseline and later antibody titres. In multivariable linear regression models adjusted for age, sex, ethnicity and immune status, higher baseline viral load was associated with a higher antibody titre at one, three and six months (Fig. 2).

**Fig. 3:**
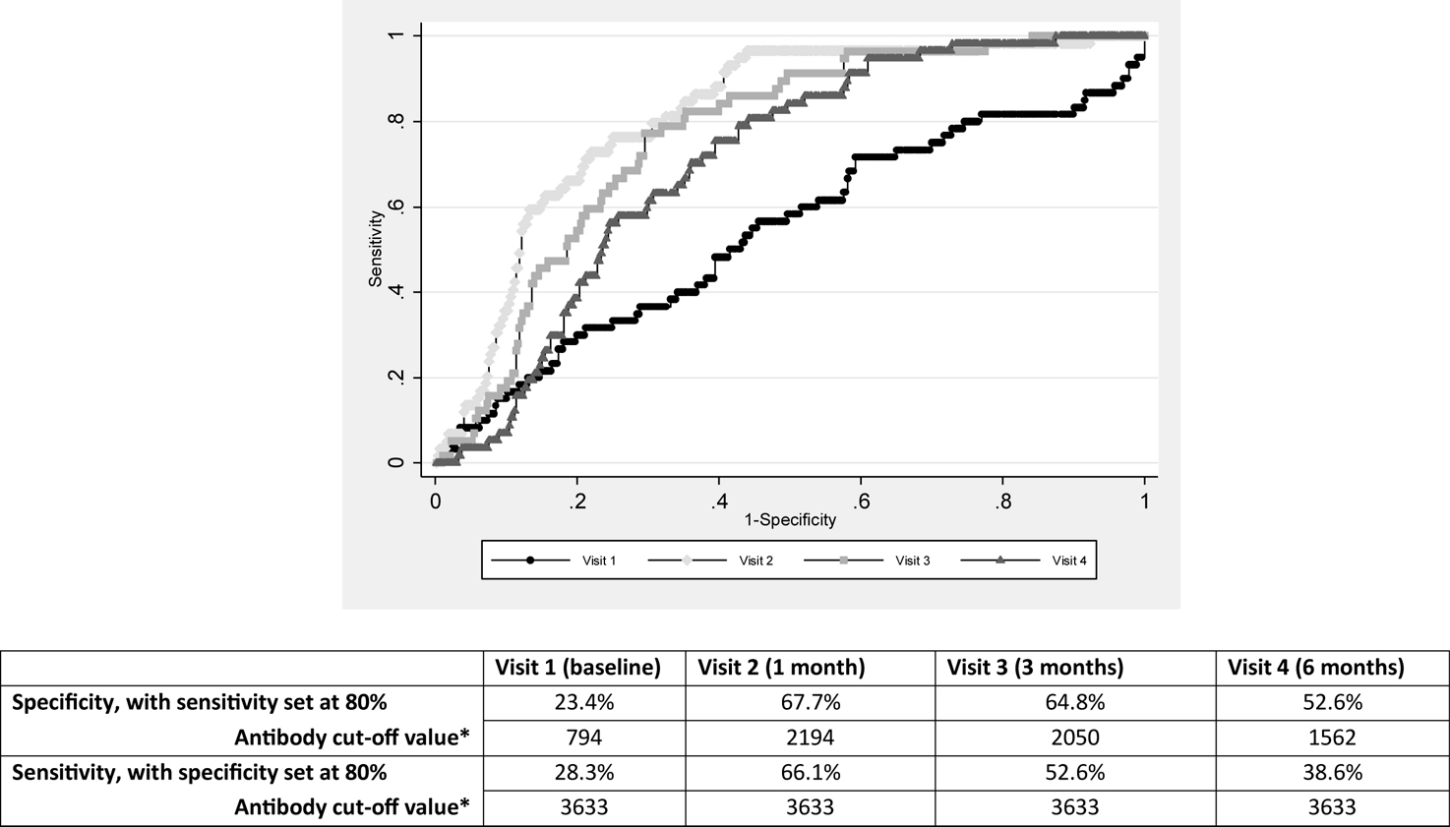
ROC curve with table showing antibody cut-off values and specificity/sensitivity if test was required to have 80% sensitivity/specificity, at each visit. *If the VZV IgG antibody titre value is greater than or equal to the cut-off, then the individual is declared positive (affected) by this cut-off approach, else negative (healthy), and these are compared to the actual status to determine sensitivity and specificity.

Antibody titre was higher in shingles patients at 1, 3 and 6 months from baseline, compared to controls; median log antibody titre was 3.16 (IQR: 2.92–3.45) among controls. ROC analysis (Fig. 3) demonstrated that to achieve 80% sensitivity, specificity would be 23.4%, 67.7%, 64.8% and 52.6%, whilst to achieve 80% specificity, sensitivity would be 28.3%, 66.1%, 52.6% and 38.6% at baseline, visit 2, 3 and 4 respectively. The best obtainable specificity, at 90% sensitivity, was 59%, and the best obtainable sensitivity, at 90% specificity, was 39% (data not shown).

## 5 Discussion

We showed that baseline, rather than subsequent viral load was the strongest predictor of antibody titre at one, three and six months after an acute shingles episode. Antibody titres remained persistently elevated in shingles patients compared to healthy blood donors for at least six months, with the greatest discrimination between groups occurring at one month post shingles. Antibody titres could discriminate patients with recent shingles from healthy controls, however there was a significant trade-off between sensitivity and specificity.

Reactivation of latent VZV is largely kept in check through cell-mediated immunity [9], with antibodies playing very little role in VZV control. Individuals with severe clinical VZV reactivation including those who develop post-herpetic neuralgia often have high antibody titres, which are believed to correlate with more widespread VZV replication [10]. Our findings are consistent with this hypothesis. The lack of association found between viral loads at one, three and six months and antibody titres at the same and subsequent time points suggests that persistence of serum VZV DNA after shingles may be a function of decay rather than ongoing replication, although this finding needs to be tested in other larger populations.

Antibody titre cut-off values could be used to identify patients with shingles 1–6 months previously, but with a large trade-off between sensitivity and specificity. Whether researchers choose to set cut off values to achieve a high sensitivity e.g. when using antibody titre as an initial screening test for recent shingles, or to be highly specific e.g. in a test aimed at diagnostic confirmation, will depend on the nature and context of their research.

This study was limited by relatively small numbers of patients. Data on other potential confounding factors such as ethnicity and immune status in blood donors was lacking, so only age and sex were accounted for in the shingles patient-blood donor analysis. Nevertheless, as these factors were not associated with antibody titre in shingles patients, results are unlikely to have been notably affected.

In conclusion, there is evidence for endogenous boosting of VZV antibody levels by clinical VZV reactivation and the level of boosting is dependent upon baseline viral replication. Additionally, antibody titres could discriminate post-shingles patients from healthy controls, although whether to prioritise specificity or sensitivity would depend on the study question.

## Conflicts of interest

All authors report no conflicts of interest.

## Ethical approval

Ethical approval was given by East London and the City Health Authority Research Ethics Committee (Ref: LREC(R&WF) 2002/38).
